# Values Determine the (In)Effectiveness of Informational Interventions in Promoting Pro-Environmental Behavior

**DOI:** 10.1371/journal.pone.0083911

**Published:** 2013-12-18

**Authors:** Jan Willem Bolderdijk, Madelijne Gorsira, Kees Keizer, Linda Steg

**Affiliations:** 1 Department of Marketing, University of Groningen, Groningen, Groningen, The Netherlands; 2 Department of Psychology, University of Groningen, Groningen, Groningen, The Netherlands; University of St Andrews, United Kingdom

## Abstract

Informational interventions (e.g., awareness campaigns, carbon footprint calculators) are built on the assumption that informing the public about the environmental consequences of their actions should result in increased pro-environmental intentions and behavior. However, empirical support for this reasoning is mixed. In this paper, we argue that informational interventions may succeed in improving people’s knowledge about the negative environmental consequences of one’s actions, but this knowledge will not gain motivational force if people do not consider protecting the environment an important personal value. In an experiment, we measured individual differences in value priorities, and either presented participants a movie clip that portrayed the negative environmental consequences of using bottled water, or a control movie. As predicted, we found that the environmental movie improved recipients’ knowledge of the negative environmental impact of bottled water, but this knowledge only resulted in concomitant changes in intentions and acceptability of related policies among participants who strongly endorsed biospheric (i.e. environmental) values, while having no effect on those who care less about the environment. Interestingly, the results suggest that although informational interventions are perhaps not always successful in directly affecting less environmentally-conscious recipients, they could still have beneficial effects, because they make those who strongly care about the environment more inclined to act on their values.

## Introduction

“Did you know that the use of bottled water harms the environment”? Such pieces of information are often offered in attempts to persuade consumers to cease their environmentally-harmful behaviors. The underlying rationale for implementing informational interventions is intuitively appealing: since people will likely fail to act pro-environmentally when they are unaware that their behavior has a detrimental impact on environmental quality, an obvious approach would be to provide the general public with information about the negative environmental impact of their behavior, for instance through prompts and warnings, or, more recently, labeling and carbon footprint calculators [1] [2][3].

Research indeed suggests that consumers may not realize whether - and to what extent - their specific behaviors negatively affect environmental quality [4][5][6]. Moreover, people are more likely to engage in pro-environmental behavior when they are aware of the problems associated with their behavior and when they believe they can personally do something to counteract the problem [7][8][9]. Consequently, it seems logical that interventions delivering *impact information* - factual information regarding the negative environmental impact of one’s behavior [10] - could bring about greener conduct. 

Studies examining the effects of environmental information provision provide however limited support for this proposition. Some research suggests that environmental information could make people more knowledgeable [11] and - especially in combination with other measures [12] - may eventually spur more pro-environmental choices [13,14]. Other studies however concluded that the influence of environmental information on behavior, if at all, is weak [11][15][16][17]. A closer examination suggests that information is effective under specific conditions only. Information may be effective changing behaviors when such change is convenient, easy, or just not costly [18]. Moreover, information seems more effective when it is properly positioned, delivered frequently and in close proximity to the target behavior [12][19]. However, everyday experience suggests that even in these more ideal cases, there are still people who are not affected by information provision. Why is this the case? 

The answer to this question may lie in the *motivation* present within the targeted audience. Schultz [10][20] has suggested that knowledge on why (‘impact knowledge’) and how (‘procedural knowledge) consumers should engage in specific actions, in and of itself, is not motivating. Rather, lack of such knowledge is one potential barrier preventing behavior change. Knowledge is thus better viewed as necessary, but in itself insufficient condition for change. This perspective may also explain why informational interventions have been successful in educating people, but have a poor record of actually changing their behavior [21][22] – recipients are not necessarily motivated to act on environmental information [11]. 

In sum, informational interventions may only translate to behavior when the person possessing the knowledge is motivated to do something with this knowledge [20]. But what determines whether people are motivated to act upon specific types of knowledge? This paper empirically examines one potential source of such motivation – values. Specifically, building on prior research [23][24], we argue that impact information will only spur relevant actions *when recipients value environmental quality* – when they consider protecting the environment an important personal goal in their life. In other words, we argue that the effectiveness of impact information depends on the extent to which recipients endorse biospheric values [25–29]. We explain and test this reasoning in the context of environmental awareness campaigns.

Environmental awareness campaigns are often used to promote desirable behavior (e.g., refusing plastic bags) and, perhaps even more importantly [30], create support for environmental policies (e.g., a ban on plastic bags). These campaigns typically explicate the negative environmental consequences of behaviors that people consider convenient and comforting (e.g. “Plastic bags are polluting our oceans”). Such impact information could elicit cognitive dissonance [31] if recipients personally care about environmental quality, as it highlights there is a discrepancy between what they freely choose to do (viz., accepting plastic bags at the supermarket), and what they find personally important (viz., caring about the environment). Consequently, awareness campaigns could make those who care about environmental quality inclined to act on their *values* – in this case by refusing plastic bags at the cash register, or by supporting anti-plastic bag legislation. However, not everybody endorses these values [24]. People who do not strongly value environmental quality may therefore respond differently to such information: they are less likely to experience a discrepancy between their values and behavior, and can therefore afford to keep using those convenient plastic bags while still feeling good about themselves. Hence, people with weak biospheric values may not be motivated to act upon environmental information. In fact, recent research has illustrated that environmental information could even *reduce* the propensity to act pro-environmentally among recipients who do not want to be associated with environmental concern [23].

In sum, we expect that increasing knowledge about the environmental consequences of one’s actions will only translate into concomitant changes in intentions and policy acceptability if people strongly value nature and the environment. In operational terms, we expect that the effect of impact information will be moderated by biospheric value strength. 

### Current research

We conducted an experiment among a sample of Dutch citizens to test this reasoning. If provision of environmental information has any effect, it should be most pronounced for topics people know relatively little about, and for behaviors that are not bound by external constraints [12]: behaviors that are relatively costless and convenient to change. A pilot study indicated that most people are unaware of the negative environmental consequences of using bottled instead of tap water. Moreover, tap water is cheaper than bottled water, readily available, and – in countries such as the Netherlands – at least as healthy as bottled water [32]. We therefore reasoned that bottled-water usage would be a suitable topic to study, offering a realistic possibility for information provision to actually improve knowledge levels and affect (easy to change) intentions and policy support. Consequently, we tested how information regarding the negative environmental consequences of bottled water would affect recipients’ knowledge levels as well as their intentions to reduce bottled water usage and acceptability of policies aimed at reducing bottled-water consumption. We expected that watching a movie on the negative consequences of using bottled water (“environmental movie”) would lead to more knowledge about the negative impact of water bottle usage than a control movie. Yet, we expected this knowledge would only affect intentions and acceptability judgments of recipients who strongly endorse biospheric values, while having no effect on the intentions and acceptability ratings of those with weak biospheric values. In other words, we expected that biospheric value strength would moderate the effect of impact information on intentions and acceptability.

## Methods

### Participants and procedure

At the end of 2011, an internet-based experiment was published on a website that manages online questionnaires and research panels (www.ThesisTools.com). ThesisTools emailed invitations to their panel members. Panel members are matched to the Dutch population in terms of region, education, age and gender. They participate on a voluntary basis, and can stop participation at any given moment. Respondents who chose to participate received a token amount of 71 euro cents for completing the questionnaire. The University of Groningen Psychology Ethics Committee approved this study and – given the fact that fully disclosing the purpose of the study beforehand could have altered participants’ answers - waived the need for obtaining written consent from participants. The total questionnaire took respondents about 20 minutes to complete. 

In total, 266 participants started the questionnaire. Our final sample was limited to 192 adult participants who completed all parts of the questionnaire (82 males, 98 females, 12 participants did not disclose their gender). The age of participants ranged from 17 to 83 years with a mean age of 50.6 years (SD = 15.74, this range does not include 4 participants who provided highly implausible age estimates). The sample was indeed comparable to the Dutch population in terms of gender and age. People with higher education and income levels were somewhat overrepresented. 

The study started by explaining the topic (“bottled water”) and the procedure. Participants completed a measure that gauged individual differences in biospheric value strength. After that, some participants (N=99) watched a movie that displayed the harmful environmental consequences of using bottled water, while a control group of participants (N=93) viewed a neutral movie that displayed unrelated information. Afterwards, all participants filled out a questionnaire which, respectively, contained questions probing their bottled water-related intentions and acceptability rating, bottled-water related beliefs, a knowledge test and a battery of socio-demographic variables (see below). The questionnaire included some additional items (e.g. on sustainable driving styles) that are beyond the scope of this paper.

### Materials and design

Participants were randomly allocated to view either the control or environmental movie. Besides explicating the negative environmental consequences of bottled water (e.g. “water from bottles inflicts 500 times the environmental damage as water from the tap” and “there are 2 to 3 liters of water required to produce 1 liter of bottled water”), the 7-minute environmental movie (available at www.uitzendinggemist.nl/afleveringen/1178714#00:00:27) also portrayed general information (“the parliament is in favor of limiting bottled water use” and “yearly, water bottles account for millions of tons of waste”). The control movie (available at www.rug.nl/corporate/nieuws/adamsAppel/archief2010/afl08_2010) reviewed media influence and the human ability to process information. It was similar in length, but contained no references to water bottles.

*Values* were assessed by means of a validated value questionnaire comprising 16 items reflecting egoistic, hedonic, altruistic and biospheric values [33]. Given our reasoning that information about the environmental impact of bottled water would only cause dissonance among people who strongly care about environmental quality, we were specifically interested in the moderating role of biospheric values. Four items measured biospheric value strength (i.e., preventing pollution, respecting the earth, unity with nature, and protecting the environment). We also measured egoistic, hedonic and altruistic values, see prior work [33] for a description of these items. Respondents rated the importance of these values as “a guiding principle in their lives” on a nine-point scale, ranging from −1 “opposed to the value”, 0 “not at all important” to 7 “of supreme importance”. Mean scores were computed on items included in the biospheric value scale (α = .79; *M* = 5.1, *SD* = 1.20, Min = 1.3, Max = 7.0). 

*Intentions* to avoid using bottled water were measured by four items (“I’m planning on limiting my use of water bottles whenever possible”, “I’m planning on reusing water bottles”, “I’m planning on drinking tap water instead of bottled water”, “I’m planning on ordering tap water instead of bottled water in restaurants”) on a 7-points Likert scale (1 “completely disagree” – 7 “completely agree”). The average score across the four items were computed, which formed an internally reliable scale (α = .79; M = 5.7, SD =1.25). 

*Acceptability*. Participants judged the acceptability of six bottled-water related policies (“Bottled water becomes more expensive due to environmental taxation”, “Companies and educational facilities should no longer be allowed to sell bottled water, but should facilitate tap water consumption”, “The availability of bottled water is being limited”, “More locations will become available (e.g. at gas stations) for tap water, so that bottles can be refilled easily”, “Water bottles should be banned from stores”, “There will be more education about the negative environmental consequences of bottled water”). All items were scored on a 7-points Likert scale (1 “not acceptable” – 7 “very acceptable”). The average score across the items were computed, which formed an internally reliable scale (α = .83; M = 5.1, SD =1.25). 

*Beliefs.* In line with the notion that changes in intentions are preceded by changes in beliefs [34][30][7] (particularly problem awareness, outcome efficacy and feelings of moral obligation) [35], we also explored how the movie would affect people’s bottled-water related beliefs. We probed participants’ awareness of the environmental impact of bottled water (“problem awareness”), their opinions regarding the usefulness of reducing their bottled water usage (“outcome efficacy”), and their perceived moral obligation to reduce the use of bottled water (“personal norm”). Problem awareness was measured by three items (“I’m concerned about the amount of CO_2_ emissions that are caused by the use of bottled water”, “The environmental damage caused by plastic bottles is very serious”, “The transportation of plastic bottles consumes much energy”) on a 7-points Likert scale (1 “completely disagree” – 7 “completely agree”), which formed an internally reliable scale (α = .78; M = 5.0, SD =1.39). Outcome efficacy was measured by three items (“It is useful for me not to buy bottled water, as to reduce environmental damage”, “It is pointless to reduce my consumption of bottled water” (reverse scored), “I feel that buying less water bottles is useful to reduce environmental problems”) on a 7-points Likert scale (1 “completely disagree” – 7 “completely agree”), which formed an internally reliable scale (α = .73; M = 5.1, SD =1.51). Personal norm to avoid the usage of bottled water was measured by three items (“I feel morally obliged to reuse plastic water bottles”, “Buying less water bottles would make me a better person”, “I feel guilty when I fail to reuse water bottles”) on a 7-points Likert scale (1 “completely disagree” – 7 “completely agree”), which formed an internally reliable scale (α = .73; M = 4.4, SD =1.59). 

*Knowledge* was measured by asking respondent to fill out a quiz. The quiz contained 11 true/false statements that were addressed in the environmental movie (e.g. “The quality of water from plastic bottles is higher than the quality of our tap water”, “The majority of water bottles available in Dutch stores is produced in the Netherlands”). To prevent respondents from guessing the right answer, we urged respondents to choose the option “I don’t know” if they had no idea which of the answers was correct or not. Scores on the quiz ranged from 0 (none) and 11 (all) correct answers. On average, participants answered 7.1 items correctly (SD = 2.57). 

## Results

In order to examine how the environmental and control movie affected participants’ knowledge, bottled-water related intentions, acceptability ratings, and beliefs, we regressed movie content, (mean centered) biospheric value strength and their interaction term onto participants’ scores on the dependent measures introduced above, using the PROCESS procedure for SPSS [36]. For ease of reading, we summarized the multiple regression statistics in Table 1, while reporting the environmental and control movie group means throughout the text. 

**Table 1:** Regression of Movie Content, Biospheric Value Strength and their Interaction Term onto Knowledge, Intentions, Acceptability Judgments, Personal Norm, Problem Awareness and Outcome Efficacy.

		Knowledge			Intentions			Acceptability			Personal Norm			Problem Awareness			Outcome efficacy	
		*b*	*t*		*b*	*t*		*b*	*t*		*b*	*t*		*b*	*t*		*b*	*t*
Movie Content		.51	2.84**		.06	.69		.06	.72		.02	.19		.04	.38		-.05	-.49
Biospheric value strength		.10	.67		.26	3.56***		.36	5.01***		.35	3.72***		.39	4.77***		.39	4.42***
Interaction term		.30	1.89		.15	1.98*		.14	1.99*		.12	1.22		.13	1.59		.15	1.67
R^2^		.06			.10			.16			.09			.14			.13	
F		4.21**			6.80***			11.54***			5.97***			9.86***			9.01***	
N=192																		

* *p*<.05, ** p<.01, *** *p*<.001

### Effects of movie content on environmental knowledge

We first examined whether watching the environmental versus control movie affected knowledge levels, as measured by scores on the pop quiz at the end of the questionnaire (see Table 1, first column). We found a main effect of movie content: participants who saw the environmental movie (M = 7.6, SD = 2.95) scored higher on the knowledge quiz than participants who saw the control movie (M = 6.6, SD = 1.98), indicating that the environmental movie had the intended educational effect. Interestingly - and in line with some recent findings [37] - we found no main effect of biospheric value strength: people who care much about environmental quality were not necessarily more knowledgeable about the negative environmental consequences of using bottled water than people who care less about environmental quality. 

### Effects of movie content on intentions and acceptability judgments

Employing the same multiple regression analysis, we subsequently examined how information about the environmental impact of bottled water affected intentions to avoid using bottled water and acceptability judgments, respectively (see Table 1, second and third column). When focusing on the sample as a whole, it appears that the environmental movie did not strengthen intentions: participants who saw the environmental movie (M = 5.8, SD = 1.30) did not report significantly stronger intentions to reduce using bottled water than those who saw the control movie (M = 5.7, SD = 1.21). The environmental movie also did not seem to boost acceptability judgments either: people who saw the environmental movie (M = 5.2, SD = 1.33) expressed about as much support as those who saw the control movie (M = 5.1, SD = 1.18). We did find a significant main effect of biospheric value strength on both variables: participants who strongly endorse biospheric values expressed stronger intentions to reduce the use of bottled water, and were more supportive of policies that discourage the use of bottled water, regardless of whether they just viewed the environmental or control movie. 

Based on these main effects, one could be tempted to conclude that the environmental movie had no motivational effect: intentions and acceptability judgments seem primarily driven by values, not by the type of movie participants saw. However, as argued in the introduction, the effect of information on behavior may depend on people’s endorsement of biospheric values. Indeed, we found significant interaction effects between movie content and biospheric value strength on intentions and acceptability (see Table 1, second and third column), suggesting that the effect of movie content depended on biospheric value strength. Note that although we were specifically interested in the moderating role of biospheric values, we also tested whether altruistic, egoistic and hedonic values would moderate the effect. As expected, the impact of movie content on intentions and acceptability was not affected by the strength of altruistic, egoistic or hedonic values, but only by biospheric values.

**Figure 1:**
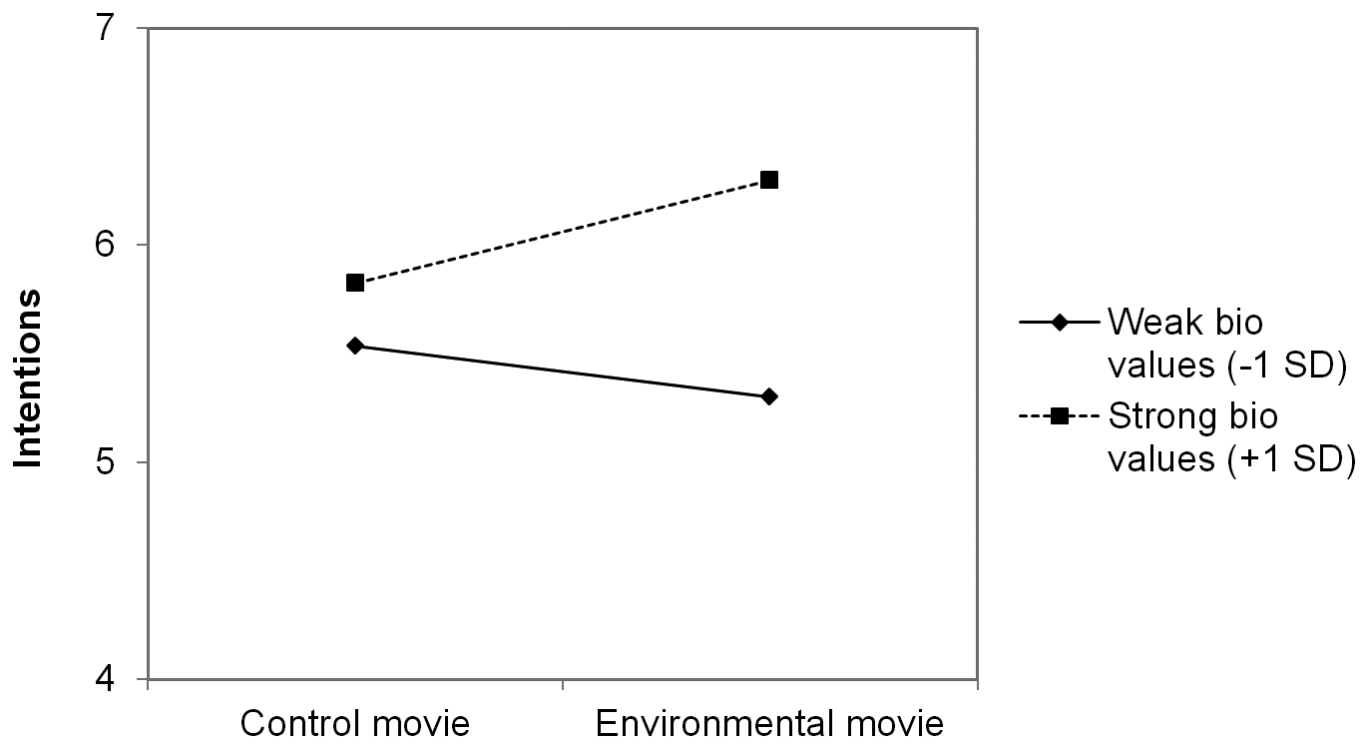
Effect of movie content and biospheric value strength on intentions to avoid the use of bottled water.

**Figure 2:**
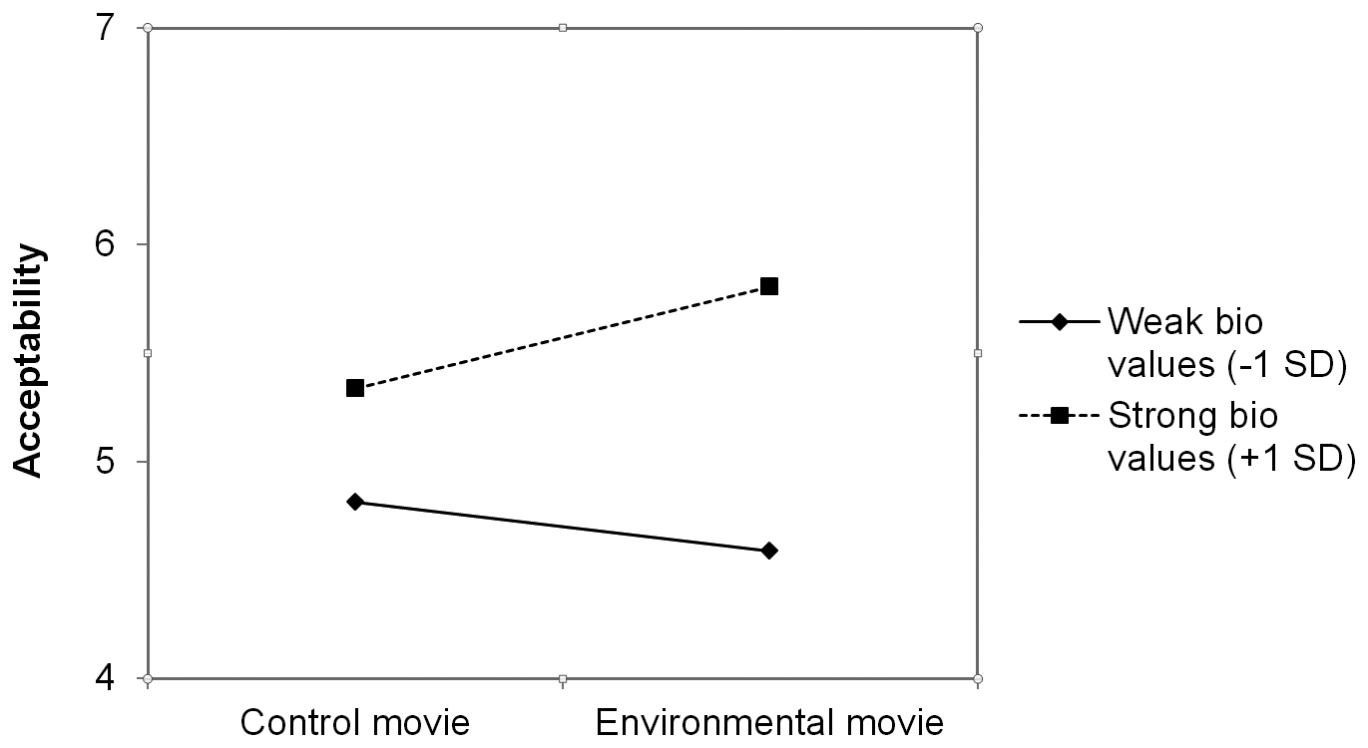
Effect of movie content and biospheric value strength on acceptability of policies aimed to limit the use of bottled water.

We next zoomed in on the significant interaction between biospheric value strength and movie content. Simple slope analyses [38] revealed that for participants with relatively strong biospheric values (+1SD above the mean), viewing the environmental movie has the intended effect: relative to participants who saw the control movie, participants with relatively strong biospheric values expressed even stronger intentions (β =.24, t(191) = 1.92, p = .05) and judged bottled water policies more positively (β =.23, t(191) = 1.95, p = .05) after viewing the environmental movie. For participants with weak biospheric values (-1SD) however, the environmental movie did not have the intended effect. If anything, the environmental movie seemed to weaken intentions (β = -.12, t(191) = -.93, n.s.) and *decrease* (β = -.11, t(191) = -.92, n.s.) acceptability judgments relative to participants who saw the control movie. This effect is visually illustrated in Figures 1 and 2, in which we plotted the effect of movie content for people with strong (+1SD) and weak (-1SD) biospheric values separately. 

To gain more insight into these interactions, we employed the Johnson-Neyman technique [39] to identify the range of biospheric values for where there was a significant simple effect of movie content on intentions and acceptability, respectively. It appears that viewing the environmental, rather than the neutral movie, only resulted in higher intentions among participants who scored relatively high (6.43 and above) on the biospheric value scale (BJN =.26, SE =.13, p =.05), and did not affect those who scored lower on the value scale. The results for acceptability judgments were similar: we only found a positive effect of movie content for people who care deeply about environmental quality, that is, people who score 6.33 or higher on the biospheric value scale (BJN = .24, SE = .12, p =.05), and not for those with weaker biospheric values. In sum, it seems that the environmental movie only affected those who strongly care about environmental issues. 

### Effects of movie content on beliefs on the use of bottled-water

We then explored how the different movies affected people’s bottled-water related beliefs, as measured by problem awareness, outcome efficacy, and respectively personal norms to reduce the use of bottled water usage (see Table 1, fourth to sixth column for results of the multiple regression analysis). The environmental movie did not seem to make people more aware of the negative environmental impact of bottled water (M = 5.0, SD = 1.51 for the environmental movie, M = 5.0, SD = 1.26 for the control movie), did not seem to convince people that their reduction of the use of bottled water would have a positive impact on the environment (M = 5.0, SD = 1.66 for the environmental movie, M = 5.1, SD = 1.35 for the control movie) and did not elicit stronger feelings of moral obligation to reduce the use of bottled water (M = 4.4, SD = 1.63 for the environmental movie, M = 4.4, SD = 1.54 for the control movie). We did however find main effects of biospheric value strength: people with stronger biospheric values were more aware of the problems caused by bottled water, were more convinced that reducing the use of bottled would have a positive impact on environmental quality, and expressed stronger feelings of moral obligation than those with weak biospheric values. Although pointing in the same direction - and in line with the effects on intentions and acceptability - we did not find statistically significant interaction effects. 

In sum, the environmental movie did not seem to significantly improve the problem awareness, outcome efficacy and personal norms of people with strong biospheric values above and beyond what they already believed. Importantly, the movie also did not alter beliefs of people with weak biospheric values. These findings therefore suggest that participants’ beliefs regarding the use of bottled water were a priori determined by values, and were not affected by the information provided in the environmental movie.

## Discussion

Given the urgency to address environmental problems, it is crucial to gain a better understanding of why some people do, and others do not respond to information about the environmental impact of their actions. The results of our experiment confirm that providing impact information, across the board, may succeed in increasing people’s knowledge regarding the negative environmental impact of their actions. However, what people in turn *do* with this knowledge seems to hinge on whether recipients personally care about environmental quality: the environmental movie used in this experiment only had the intended effect - increased intentions to reduce the use of bottled water, and more support for policies that discourage the use of bottled water - among people who strongly endorse biospheric values, while having no effect on the intentions and acceptability judgments of people with weaker biospheric values. As such, this study empirically validates the notion that impact information may only galvanize relevant action when people are motivated to do something with this information [10], as reflected in their values.

Following prior research [35], one would expect that interventions delivering impact information could spur changes in recipient’s intentions and acceptability judgments because these interventions increase people’s awareness of environmental problems and/or give people the idea that they can personally contribute to the solution of such problems. Our research found no direct support for this process: participants with weak biospheric values were not convinced of the importance of reducing bottled water, and the environmental movie did not change this. Participants with strong biospheric values, on the other hand, were convinced of the importance of reducing bottled water, regardless of whether they saw an environmental or unrelated movie. 

Based on these, as well as prior findings [40] one could be tempted to conclude that informational interventions such as these are futile, as people are unlikely to reconsider their value-driven beliefs. We did however observe the environmental movie to result in an increase in intentions and acceptability among participants with strong biospheric values. Thus, our results point to a more nuanced conclusion already alluded to by prior research [11][20]: informational interventions could potentially motivate change among groups that value the environment, but not necessarily because it convinces them to adjust their environmentally-relevant beliefs, but rather because it makes people more inclined to act on their values. 

Due to the limitations of the online environment, we could only examine the movie’s effect on participants’ self-reported statements that were recorded directly after viewing the movie. As such, we cannot be sure whether the environmental movie affected participants’ actual, long-term, use of bottled water. Still, given that the environmental movie did not even affect the intentions and acceptability ratings of people with weak biospheric values, it seems unlikely it did succeed in altering their actual consumption of bottled water. However, before drawing any definite conclusions regarding the effectiveness of informational interventions, future research including behavioral measures is needed to corroborate our results. Such research should explore whether these findings also hold for (long-term) changes in other, more difficult types of behavior. 

Despite the fact that educating people on how and why they should act pro-environmentally is perhaps the single most employed means of motivating consumers, it is to date not fully clear why some individuals do, and others do not respond to environmental information. Our study empirically demonstrates the relevance of considering individual differences in value priorities when trying to understand the effects of environmental information provision: informational interventions only seem directly effective in motivating those who care about preserving environmental quality. Our study thus offers directions for future research, as well as some important practical implications: informational interventions may become more effective when they are specifically designed and delivered to targeted groups, rather than adopting a general ‘one-size-fits-all’ strategy [23][41][42]. In a broader sense, this study also suggests that to facilitate pro-environmental behavior and beliefs across the populace at large, it may be more productive for behavioral interventions to focus on *breeding* and *activating* biospheric values, rather than relying on the assumption that providing information per se will be sufficient to accomplish behavior change.
